# Challenges in malaria modeling

**DOI:** 10.1186/1475-2875-13-S1-O14

**Published:** 2014-09-22

**Authors:** F Ellis McKenzie

**Affiliations:** 1 Fogarty International Center, National Institutes of Health, Bethesda, USA

## 

Mathematical models can be useful tools in projects across the entire range of malaria research and intervention. The past decade has seen significant progress in malaria modeling, but challenges remain in applying, testing and refining current tools and in developing new ones.

